# Intravenous Metoclopramide to Improve the Success Rate of Blind Bedside Post-pyloric Placement of Feeding Tube in Critically Ill Children: A Randomized, Double-Blind, Placebo-Controlled Study

**DOI:** 10.3389/fped.2021.739247

**Published:** 2021-12-22

**Authors:** Sirima Ketsuwan, Pornthep Tanpowpong, Nichanan Ruangwattanapaisarn, Supatra Phaopant, Nattanicha Suppalarkbunlue, Chula Kooanantkul, Nattachai Anantasit, Jarin Vaewpanich

**Affiliations:** ^1^Division of Pediatric Critical Care Medicine, Department of Pediatrics, Faculty of Medicine, Ramathibodi Hospital, Mahidol University, Bangkok, Thailand; ^2^Division of Gastroenterology, Department of Pediatrics, Faculty of Medicine, Ramathibodi Hospital, Mahidol University, Bangkok, Thailand; ^3^Department of Diagnostic and Therapeutic Radiology, Faculty of Medicine, Ramathibodi Hospital, Mahidol University, Bangkok, Thailand; ^4^Department of Nursing, Faculty of Medicine, Ramathibodi Hospital, Mahidol University, Bangkok, Thailand; ^5^Clinical Pharmacy Department, Faculty of Medicine, Ramathibodi Hospital, Mahidol University, Bangkok, Thailand; ^6^Super Kids Center, Vejthani Hospital, Bangkok, Thailand

**Keywords:** post-pyloric feeding, metoclopramide, blind bedside placement, nasojejunal feeding, critically ill children

## Abstract

**Objective:** Impaired gastric emptying is a common cause of delayed feeding in critically ill children. Post-pyloric feeding may help improve feeding intolerance and nutritional status and, hence, contribute to a better outcome. However, post-pyloric feeding tube insertion is usually delayed due to a technical difficulty. Therefore, prokinetic agents have been used to facilitate blind bedside post-pyloric feeding tube insertion. Metoclopramide is a potent prokinetic agent that has also been used to improve motility in adults and children admitted to intensive care units. The objective of this study was to determine the efficacy of intravenous metoclopramide in promoting the success rate of blind bedside post-pyloric feeding tube placement in critically ill children.

**Design:** The design of this study is randomized, double blind, placebo controlled.

**Setting:** The setting of the study is a single-center pediatric intensive care unit.

**Patients:** Children aged 1 month−18 years admitted to the pediatric intensive care unit with severe illness or feeding intolerance were enrolled in this study.

**Intervention:** Patients were randomly selected to receive intravenous metoclopramide or 0.9% normal saline solution (the placebo) prior to the tube insertion. The study outcome was the success rate of post-pyloric feeding tube placement confirmed by an abdominal radiography 6–8 h after the insertion.

**Measurements and Main Results:** We found that patients receiving metoclopramide had a higher success rate (37/42, 88%) of post-pyloric feeding tube placement than the placebo (28/40, 70%) *(p* = 0.04). Patients who received sedative drug or narcotic agent showed a tendency of higher success rate (*p* = 0.08).

**Conclusion:** Intravenous metoclopramide improves the success rate of blind bedside post-pyloric placement of feeding tube in critically ill children.

**Trial Registration:** Thai Clinical Trial Registry TCTR20190821002. Registered 15th August 2019.

## Introduction

Malnutrition is a common problem in critically ill patients causing increased morbidity and mortality (1, 2), and enteral feeding is the preferred route of nutritional support to improve nutritional status for most critically ill patients with an adequate gastrointestinal function (1, 3). However, some children cannot tolerate intragastric feeding due to delayed gastric emptying, impaired motility, or carry a higher risk of aspiration or severe gastroesophageal reflux, which can cause feeding postponement. Therefore, post-pyloric feeding may be preferred for patients at high risk for aspiration and feeding intolerance (4, 5). Previously, fluoroscopic and endoscopic procedures were used in the post-pyloric tube placement, but the procedures are costly, increase radiation exposure, and require a transfer of patient to the interventional radiology or endoscopy suites (6–8).

Blind bedside post-pyloric feeding tube placement has been shown to be safe and feasible for early enteral feeding in critically ill patients (5, 9). Some studies have suggested a benefit of using a motility agent in the placement, but a definitive study, such as an RCT, has not been completed (10, 11). Metoclopramide, a prokinetic agent, works by blocking dopaminergic receptor and increasing gastric motility. It is used to treat nausea and vomiting in several conditions such as post-surgery, gastroesophageal reflux, and chemotherapy-induced vomiting (12). Studies in adults demonstrated promising results when using metoclopramide to improve a success rate of tube insertion (13, 14), but data in children are limited. Therefore, we aimed to determine whether intravenous metoclopramide improved the success rate of blind bedside post-pyloric placement of feeding tube in critically ill children in a prospective, randomized, double-blind, placebo-controlled fashion.

## Materials and Methods

### Study Design

We conducted a prospective, randomized, double-blind, placebo-controlled study in the Pediatric Intensive Care Unit (PICU) at a tertiary care teaching hospital. The study was approved by the Committee on Human Rights Related to Research Involving Human Subjects, Faculty of Medicine Ramathibodi Hospital, Mahidol University, and written informed consent was obtained from each patient or their legal guardians. This trial study was registered at the Thai Clinical Trial Registry (TCTR20190821002).

### Patients

Patients admitted to the PICU between December 2018 and January 2020 with the following inclusion criteria were included: critically ill, aged 1 month to 18 years, required enteral nutrition, and having severe illness or feeding intolerance. Patients having a major abdominal surgery, a known history of malrotation, an active upper gastrointestinal bleeding, severe coagulopathy, or allergic to metoclopramide were excluded. The decision on commencing the enteral feeding was made by the on-service attending physician.

**Figure 1:**
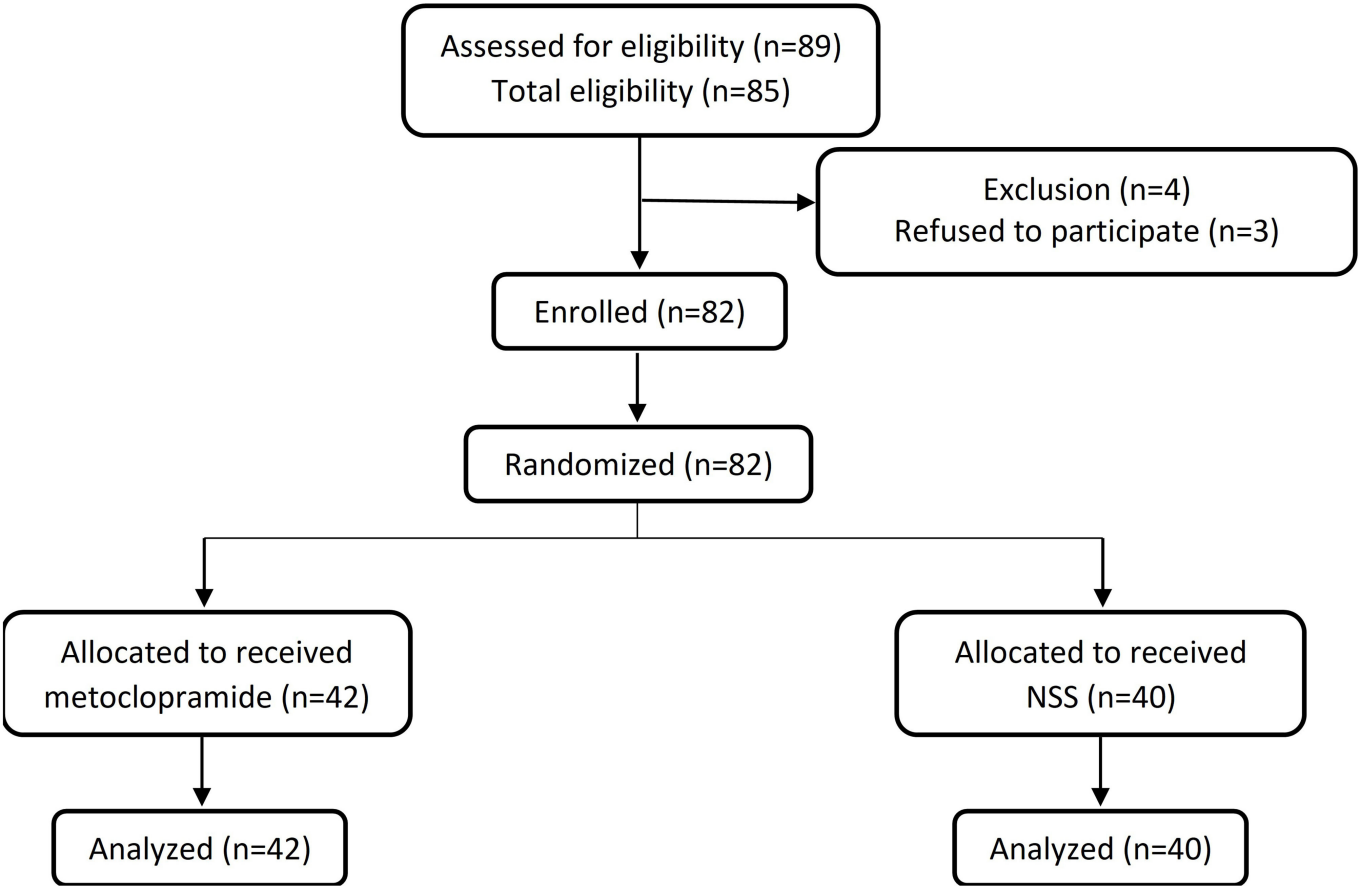
Consort diagram of the study.

In Figure 1, eligible patients who fulfilled the inclusion criteria were randomly allocated by a computer-generated block-of-four randomization and assigned to receive either intravenous metoclopramide (the metoclopramide group) or 0.9% normal saline solution (the placebo group) of similar physical appearance. Clinicians who treated the patients and an investigator (SK) who inserted the feeding tube were not aware of the allocation. All the randomization, the allocation, and the medication preparation were the responsibility of a pharmacist.

### Intervention

Patients in the metoclopramide group received 0.1 mg/kg of metoclopramide intravenously 30 min before the feeding tube insertion, and in the placebo group, 0.9% normal saline solution was given. The tube used was radiopaque unweighted silicone tube without wire stylet (Fortune Medical Instrument, Taiwan). The length of the nasointestinal tube before insertion was measured following the method of a previous study (15). The measurement started from the nose to one ear and then to the mid-point between the xiphisternum and the umbilicus to obtain the length for tube position in the stomach. Then, the measurement was continued to the right iliac crest to get a final length for insertion. When the measurement was done, the tube was lubricated with sterile gel and inserted from the nostril to the stomach in a supine position, with the head tilted at 30° elevation. The position of the inserted tube was confirmed by injecting air into the stomach. Then, the patient was turned to his/her right side down onto the bed, and the tube was pushed down to the pre-measured length with a corkscrew technique and fixed onto the patient's nose. The patient remained on the same position for at least 3 h after the insertion. All steps of feeding tube insertion were performed only once by one experienced physician.

### Data Collection and the Study Outcome

Demographic characteristics, primary diagnosis, and indication admitted to the PICU, disease severity, Pediatric Risk of Mortality III (PRISM III), and Pediatric Logistic Organ Dysfunction (PELOD) scores, medications, and potential adverse events from the medication including extrapyramidal side effects, life-threatening arrhythmia, or drug allergy were recorded. Besides, adverse events from the tube insertion including epistaxis, vomiting, and bowel perforation were also recorded. An abdominal radiograph was done to evaluate the feeding tube position at 6 to 8 h after the insertion, and the position was confirmed by a pediatric radiologist who was also blinded to this study. The study outcome was a successful placement of feeding tube into the post-pyloric area (duodenum and proximal jejunum).

### Statistical Analysis

The sample size was calculated by the Power and Sample size Calculation Program version 3.1.2 using type I error of 0.05 and power of 80%. Based on previous studies, 82 patients were required to show an increased success rate from 40 to 81% using prokinetic agent administration (16, 17).

The demographic data were analyzed by independent sample *t*-test or Mann–Whitney *U*-test. Position of the feeding tube was compared between groups using Chi-square test. Bivariate analysis was performed to study the impact of sedative and inotropic drugs. The effective size of metoclopramide was calculated by using odds ratio. The data were analyzed using SPSS program version 24.

## Results

Eighty-two patients (42 in the metoclopramide group and 40 in the placebo group) were enrolled in the study. The demographic data of the patients are presented in Table 1. There were no significant differences of baseline characteristics, mechanical ventilation, and drug administered between the two groups. In addition, the PICU length of stay, the total hospital stay, the PRISM III, and the PELOD were not significantly different (Table 2).

**Table 1:** Demographic and clinical characteristics of the metoclopramide and the placebo groups.

**Characteristics**	**Metoclopramide (*N* = 42)**	**Placebo (*N*= 40)**	***p* value**
Males; n (%)	21 (50)	23 (58)	0.50
Age [months]; median (IQR)	21 (5, 116)	17 (3, 55)	0.48
Body weight; median (IQR)	11 (6, 21)	10.5 (5, 16)	0.56
Primary diagnosis; n (%)			0.27
Neurological	10.0 (24)	7 (18)	
Cardiovascular system	7 (17)	8 (20)	
Respiratory system	18 (43)	17 (43)	
Gastrointestinal system	3 (7)	0 (0)	
Others	4 (9)	8 (19)	
Respiratory support; n (%)			0.10
Low flow oxygen cannula	3 (7)	2 (5)	
HHHFNC^a^	15 (36)	6 (15)	
CPAP^b^/BIPAP^c^	1 (2)	1 (3)	
Mechanical ventilator	23 (55)	31 (77)	
Mechanical ventilator day (days); median (IQR)	11 (3, 21)	14 (6, 28)	0.10
Procedure was done in PICU; n (%)	34 (81)	31 (78)	0.70
Procedural time; median (IQR)	10 (7, 10)	10 (7, 10)	0.82
Feeding; n (%)			0.20
Absolute nil per os	1 (2)	4 (10)	
Partial feeding	41 (98)	36 (90)	
Feeding intolerance or severe reflux	14 (33)	11 (28)	0.57
Use of inotrope; n (%)	15 (36)	11 (28)	0.42
Use of Muscle relaxant; n (%)	6 (14)	6 (15)	0.93
Use of sedative drug; n (%)			
Continuous drip	20 (48)	18 (45)	0.81
Intermittent dose	20 (48)	26 (65)	0.11

a *Heated humidified high-flow nasal cannula*.

b *Continuous positive airway pressure*.

c *Bilevel positive airway pressure*.

**Table 2:** Severity, mortality, length of pediatric intensive care unit (PICU) stay, and length of hospital stay.

**Characteristics**	**Metoclopramide (*N* = 42)**	**Placebo (*N* = 40)**	***p* value**
PRISM^a^; mean (SD)	8 (7)	8 (6)	0.71
PELOD^b^; mean (SD)	5 (4)	5 (4)	0.41
28-day mortality; n (%)	8 (19)	8 (20)	0.91
Length of PICU stay (days); median (IQR)	11 (5, 21)	12 (6, 26)	0.22
Length of hospital stay (days); median (IQR)	24 (13, 42)	30 (21, 55)	0.14

a *Pediatric Risk of Mortality III score*.

b *Pediatric Logistic Organ Dysfunction score*.

The mean insertion time was 9.5 ± 3.6 min. The metoclopramide group had a higher success rate of post-pyloric feeding tube insertion than the placebo group [88 vs. 70%, odds ratio 3.2 (95% CI: 1.0, 10.0), *p* = 0.04, Table 3]. Patients who received sedative drug or narcotic agent were more likely to have a successful tube insertion (*p* = 0.08, Table 4). Other factors, such as the use of inotropic drugs, PRISM III score ≥10, demonstrated no statistical significance. No serious adverse events related to the medication or tube placement were encountered.

**Table 3:** Success rate of post-pyloric feeding tube placement compared between the metoclopramide and the placebo groups.

**Feeding tube position**	**Metoclopramide (*n* = 42)**	**Placebo (*n* = 40)**	***p-*Value**
Post-pyloric^a^: n (%) • D1^b^: n (%) • D2^c^: n (%) • D3^d^: n (%) • D4^e^: n (%) • Proximal jejunum^f^: n (%)	37 (88) 4 (9) 10 (24) 4 (9) 5 (12) 14 (33)	28 (70) 4 (10) 7 (18) 2 (5) 1 (2) 14 (35)	0.04

a *Reaching the duodenal bulb and beyond*.

b *Reaching the duodenal bulb to the first portion of the duodenum*.

c *Reaching the first portion of the duodenum to the second portion of the duodenum*.

d *Reaching the second portion of the duodenum to the third portion of the duodenum*.

e *Reaching the third portion of the duodenum to the fourth portion of the duodenum*.

f *Reaching the proximal jejunum or beyond*.

**Table 4:** Factors influencing the success rate of post-pyloric position of feeding tube.

**Parameter**	**Gastric position (*n* = 17)**	**Post-pyloric position (*n* = 65)**	***p-*Value**
Sedative drug or narcotic agent	9 (53%)	49 (75%)	0.08
Inotropic use	3 (18%)	11 (17%)	>0.99
PRISM III^a^ ≥ 10	7 (41%)	26 (40%)	0.93

a *Pediatric Risk of Mortality III score*.

## Discussion

To our knowledge, this is the first randomized, double-blind, placebo-controlled trial demonstrating that the intravenous metoclopramide before feeding tube insertion could improve the success rate of blind bedside post-pyloric tube placement in critically ill children. As a selective dopamine-2 receptor antagonist and a 5-hydroxytryptamine receptor 4 agonist (18), metoclopramide helps promote gastric emptying and enhances cholinergic-induced peristaltic contractility of the stomach. Metoclopramide has been used to improve peristalsis and facilitate post-pyloric tube placement (10, 19, 20). A randomized controlled study in adults showed the increased success rate of post-pyloric tube placement of 55% in the metoclopramide group compared with 27.3% in the placebo group (13). Nevertheless, a systematic review of four studies demonstrated that metoclopramide did not improve the chance of success (RR 0.82; 95% CI 0.61, 1.10) (21). However, the study on this regard in children is scarce.

Our study showing 88% success rate of blind bedside post-pyloric tube placement in the metoclopramide group, compared with 70% in the placebo group, supports the use of metoclopramide in pediatric post-pyloric tube placement. While a previous study reported successful tube placement of 38% among the non-intervention standard group (22), our study demonstrated a higher success rate; the increase in the success rate may be contributed by the experienced tube placement operator (9). Some studies mentioned about the training years of physicians being the variable associated with successful feeding tube placement (11, 15); therefore, the tube placement in this study was performed by one single clinician to limit this confounder. There were no significant differences in the advancement through the small intestine (Table 3), which may be due to small sample size, anatomical variation of the small bowel, and different clinical settings, e.g., degree of dysmotility.

Different techniques have been used for post pyloric feeding tube placement in pediatric patients including an electromagnetic guidance technique (22) and an insufflation air technique (23). However, the electromagnetic guidance carries a high cost and a need of specialized equipment, and the air insufflation may cause abdominal discomfort. Blind bedside post-pyloric tube placement is an alternative technique, which is considered safe, inexpensive, and effective (24). We considered the technique time-efficient as the mean insertion time was 9.5 ± 3.6 min, which was similar to a previous study (25).

Most PICU patients require sedative drug or narcotic agent. In our study, we found that both the sedative drug and the narcotic agent may enhance the success rate of tube placement (*p* = 0.08). Hence, we hypothesized that the cooperation and comfort of the patient during tube placement is crucial to the increase of success rate.

Theoretically, based on the pharmacokinetic property of intravenous metoclopramide having an average elimination half-life of 4.9 h (26), a duration of 6–8 h to perform plain abdominal radiography was applied in the present study. Furthermore, this duration was also used for the migration of feeding tube along with the bowel peristalsis. However, studies in adult patients have found that a much longer observation time of 24 to 72 h was applied after feeding tube insertion (13, 27, 28). In pediatric patients, they had less energy reserve than the adults, and the standard practice guideline also recommends early enteral feeding as soon as possible (1).

Adverse effects from intravenous metoclopramide are arrhythmias and extrapyramidal side effects (13, 26), but none was reported in our study. Additionally, the adverse events associated with nasointestinal tube insertion (such as misplacement, epistaxis, duodenal perforation, pain, or vomiting) were reported in previous studies (13, 25, 29); we did not observe any of these events in our study.

Our study had some limitations: abdominal radiography was not performed immediately after the procedure as we wanted to wait for the maximal effect of intravenous metoclopramide on bowel peristalsis. Therefore, we are not fully able to clarify whether the tube was placed in the proper location due to the original placement or the ongoing peristalsis facilitated by the metoclopramide. However, this study has some strong points of view, which are the randomized controlled fashion and the feeding tube insertion performed by one single operator. Besides, the relatively small sample size from a single tertiary center may limit the generalizability. Further multicenter studies in various acuity settings may lead to an increase in generalizability of the aforementioned findings.

## Conclusion

Intravenous metoclopramide can improve the success rate of blind bedside post-pyloric placement of feeding tube in critically ill children.

## Data Availability Statement

The original contributions presented in the study are included in the article/supplementary material, further inquiries can be directed to the corresponding author.

## Ethics Statement

The studies involving human participants were reviewed and approved by Asst. Prof. Chusak Okascharoen, Office of The Committee for Research, Faculty of Medicine Ramathibodi Hospital, Mahidol University. Written informed consent to participate in this study was provided by the participants' legal guardian/next of kin.

## Author Contributions

JV, SK, and PT contributed to conception and design of the study. NR, SP, and NS organized the database. CK performed the statistical analysis. JV and SK wrote the first draft of the manuscript. NA wrote sections of the manuscript. All authors contributed to manuscript revision, read, and approved the submitted version.

## Funding

This study was supported by a grant from the Ramathibodi Research Fund, Faculty of Medicine Ramathibodi Hospital.

## Conflict of Interest

The authors declare that the research was conducted in the absence of any commercial or financial relationships that could be construed as a potential conflict of interest.

## Publisher's Note

All claims expressed in this article are solely those of the authors and do not necessarily represent those of their affiliated organizations, or those of the publisher, the editors and the reviewers. Any product that may be evaluated in this article, or claim that may be made by its manufacturer, is not guaranteed or endorsed by the publisher.
